# Enhancing the Poor Flow and Tableting Problems of High Drug-Loading Formulation of Canagliflozin Using Continuous Green Granulation Process and Design-of-Experiment Approach

**DOI:** 10.3390/ph13120473

**Published:** 2020-12-17

**Authors:** Bjad K. Almutairy, El-Sayed Khafagy, Ahmed Alalaiwe, Mohammed F. Aldawsari, Saad M. Alshahrani, Bader B. Alsulays, Abdullah S. Alshetaili, Sultan M. Alshehri, Mohamed H. Fayed

**Affiliations:** 1 Department of Pharmaceutics, College of Pharmacy, Prince Sattam Bin Abdulaziz University, Al-kharj 11942, Saudi Arabia; e.khafagy@psau.edu.sa (E.-S.K.); a.alalaiwe@psau.edu.sa (A.A.); moh.aldawsari@psau.edu.sa (M.F.A.); sm.alshahrani@psau.edu.sa (S.M.A.); b.alsulays@psau.edu.sa (B.B.A.); a.alshetaili@psau.edu.sa (A.S.A.); 2 Department of Pharmaceutics and Industrial Pharmacy, Faculty of Pharmacy, Suez Canal University, Ismailia 41522, Egypt; 3 Department of Pharmaceutics, College of Pharmacy, King Saud University, Riyadh 11451, Saudi Arabia; salshehri1@ksu.edu.sa; 4 College of Pharmacy, Almaarefa University, Riyadh 11597, Saudi Arabia; 5 Department of Pharmaceutics and Industrial Pharmacy, Faculty of Pharmacy, Fayoum University, Fayoum 63514, Egypt

**Keywords:** canagliflozin tablet, green granulation process, design of experiment (DoE), high drug-loading

## Abstract

Maximization of drug-loading can significantly reduce the size of dosage form and consequently decrease the cost of manufacture. In this research, two challenges were addressed: poor flow and tableting problems of high-drug loading (>70%) formulation of canagliflozin (CNG), by adopting the moisture-activated dry granulation (MADG) process. In this method, heating and drying steps were omitted so, called green granulation process. A 3^2^ full-factorial design was performed for optimization of key process variables, namely the granulation fluid level (X_1_) and the wet massing time (X_2_). Granulation of CNG was carried out in the presence of polyvinylpyrrolidone, and the prepared granules were compressed into tablets. Regression analysis demonstrated the significant (*p* ≤ 0.05) effect of X_1_ and X_2_ on properties of granules and corresponding tablets, with pronounced impact of X_1_. Additionally, marked improvement of granules’ properties and tableting of CNG were observed. Furthermore, the optimized process conditions that produced good flow properties of granules and acceptable tablets were high level of granulation fluid (3.41% *w*/*w*) and short wet massing time (1.0 min). Finally, the MADG process gives the opportunity to ameliorate the poor flow and tableting problems of CNG with lower amounts of excipients, which are important for successful development of uniform dosage unit.

## 1. Introduction

In 2013, the U.S. Food and Drug Administration had approved Canagliflozin (CNG) for the management of adult patients with type-II diabetes mellitus [1]. The CNG is one of the orally acting sodium-glucose co-transporter-2 inhibitors that reduces the renal tubular reabsorption of glucose into the systemic circulation, thus decreasing plasma glucose levels in hyperglycemic patients [2]. It is possibly prescribed as a monotherapy or in combination with any of the existing antidiabetic agents, like metformin [3]. The CNG is recommended as the first add-on agent for those hyperglycemic patients for whom monotherapy of metformin is unable to efficiently control plasma glucose levels [3]. Following single oral administration of 50, 100 and 300 mg, CNG was rapidly absorbed with peak plasma concentration attained within 1–2 h. The steady-state concentrations were achieved after 4–5 days after multiple dosing. The absolute oral bioavailability of CNG was 65% with plasma protein binding of 99%. After single oral dose administration, the terminal half-life of CNG was 10.6–13.1 h, with a large volume of distribution of 119 L. The renal clearance of CNG was in the range of 1.3–1.55 mL/min for doses of 100 and 300 mg, respectively [4].

The CNG has considerable tableting challenges due to its poor flow and a high drug-loading [5]. Besides, CNG is usually utilized in combination with metformin to produce a fixed dose combination that consequently increases the tablet size and might worsen its compressibility [6]. To overcome these challenges, the wet granulation process has been used to produce drug-excipient agglomerates with adequate flow and compressibility properties [7]. However, wet granulation is a multivariate process and is complicated in terms of process control [8]. In addition, long processing times due to wetting and drying steps, loss of activity of active molecules, in-feed fluctuation through hopper and increased cost of manufacture have been found to be disadvantageous [9].

Moisture-activated dry granulation (MADG) can be explored to defeat the formulation challenges of CNG. MADG is an innovative continuous granulation technique and the entire process could be carried out in a conventional high-shear granulator; therefore, this technique is designated as a ‘‘one-pot process” [10]. Initially, all ingredients and functional additives (i.e., disintegrants and lubricants) needed for granulation and tableting are mixed in the same pot; thus, there is no need for transfer of intermediate granules to other equipment, which reduces the time of the entire process and decreases the exposure risk to highly potent drugs [11]. Additionally, MADG needs a small amount of granulation fluid (water), so that it requires neither drying nor milling. Thus, this free of heating and drying granulation process is called the green granulation method. One more merit of this technology is that there is no problem of end-point sensitivity. Therefore, the need for costly Process Analytical Technology is minimal [10].

The process of MADG can be classified into two different stages: (1) the agglomeration stage, in which drug, binder and other functional excipients are pre-mixed in a high shear granulator, followed by activation of binder with a low amount of granulation fluid (1–4% *w*/*w*) to produce granules, and (2) the moisture absorption stage, in which the moisture of the granules is absorbed by adding an absorbent material like colloidal silicon dioxide, resulting in production of dry and free-flowing granules [11,12].

Christensen et al. reported that process variables like water volume and wet massing time had a significant impact on granules’ and tablets’ properties prepared by a MADG process using a high shear mixer [13]. Takasaki et al. studied the impact of water activity on granules’ and tablets’ properties prepared using MADG techniques. They reported that the amount of water is the predominant variable for the MADG process [12]. Moravkar et al. also investigated the MADG process to develop immediate release (IR) tablets of high-dose drug formulations. They concluded that MADG is a successful technique in the development of a high-dose IR tablet with desired pharmaceutical quality attributes [10]. However, the previous studies had been carried out using ‘trial and error’ of the one factor at a time approach (OFAT). It is a classical technique applied to recognize critical factors affecting the critical attributes of the product. However, the OFAT approach ignores the interactions between the independent process variables, which also have a significant impact on the dependent responses [14].

The process of MADG is influenced by different process variables, so the design of experiment (DoE) as a part of the quality-by-design (QbD) approach was selected and preferred over the OFAT approach as it provides systematic and comprehensive parameter analysis. The main advantages of DoE over OFAT are defining effective variables, estimating the effect of independent variables on the specified response(s), recognizing interactions between variables and optimization and modeling to generate the mathematical relationship between the independent variables and dependent response(s) [15,16]. The significance of DoE as an integral part of the drug product development has been reported [6,17]. Development of high-loading tablet formulation of CNG using MADG has not been reported in the literature.

Based on these premises, the aim of the current investigation was to (1) address the poor flow and tableting problems of CNG using the continuous MADG method, (2) application of the DoE approach to evaluate the effect of key process variables of MADG and their interactions on critical quality attributes (CQAs) of intermediate granules and corresponding tablets and (3) optimization of the key process variables using the desirability function to provide CNG tablets with desired attributes.

## 2. Results and Discussion

### 2.1. Statistical and Diagnostic Analysis of the Design

**Figure 1:**
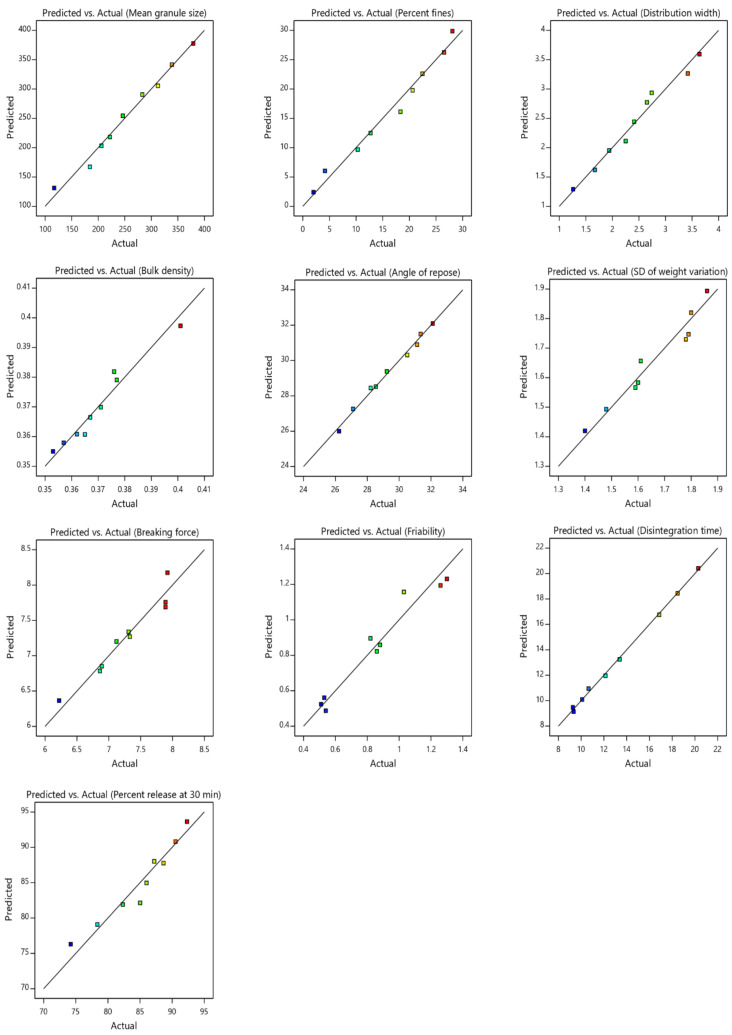
Linear correlation plot relating mean granule size, percent fines, distribution width, bulk density, angle of repose, SD of weight variation, breaking force, friability, disintegration time and drug release at 30 min, between the predicted and the measured (actual) values. SD: standard deviation.

Multiple regression analysis results of the proposed models are given in Table 1. Design-expert software can generate mathematical polynomial models (i.e., linear, 2-factor interaction, quadratic and cubic) to relate the variables to the responses. For each response, *p* < 0.05 indicates that the terms in the model reflect the behavior of the response function. In addition, the adjusted *R*^2^ of the selected model reasonably agreed with the corresponding predicted *R*^2^, with determination coefficients (*R*^2^) more than 0.8011, confirming the convenience and accuracy of the selected models [18]. Moreover, diagnostic plots were generated for granules and tablet responses to evaluate the goodness of fit of the applied model and confirm its significance. Linear correlation plots (Figure 1) between the actual and the predicted values with higher *R*^2^ indicate good model fit.

### 2.2. Influence of Process Variables on Granules Properties

#### 2.2.1. Mean Granules Size (d_50_)

**Figure 2:**
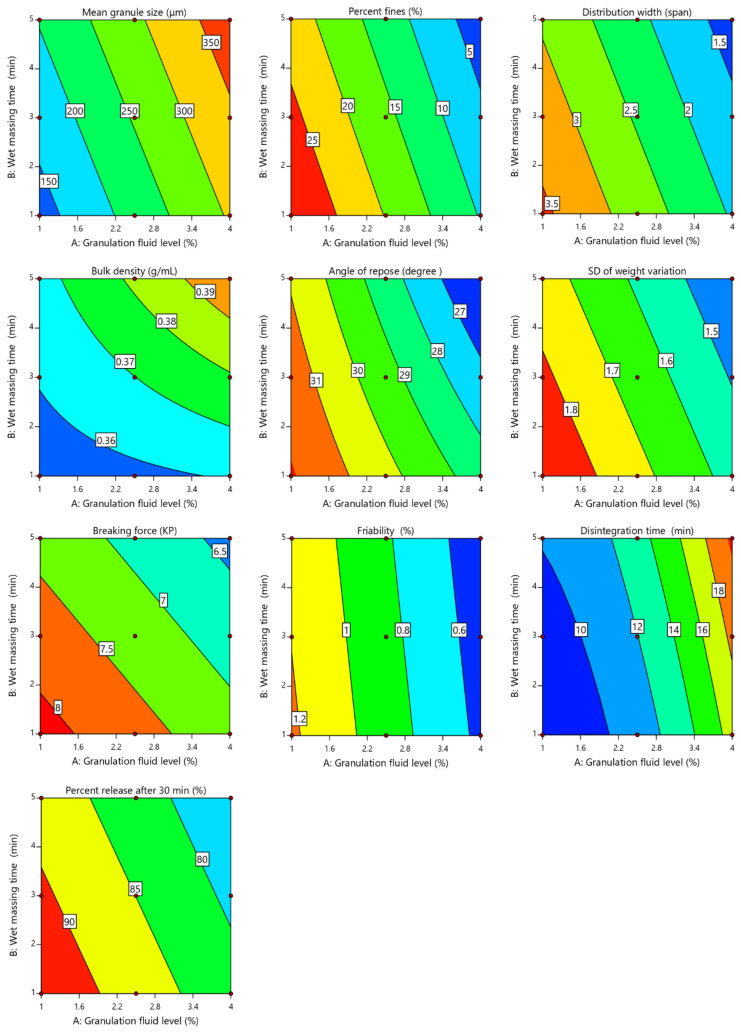
Contour plots showing the effect of the level of granulation fluid (X_1_) and the wet massing time (X_2_) on mean granule size, percent fines, distribution width, bulk density, angle of repose, SD of weight variation, breaking force, friability, disintegration time and percent release at 30 min.

As shown in Table 2, increasing the amount of granulation fluid from 1% to 4% *w*/*w* and the wet massing time from 1 to 5 min led to an increase of d_50_ from 117.12 ± 0.25 to 379.14 ± 0.33 μm, a decline in the percent fines (<50 μm) from 28.08% ± 0.013% to 2.01% ± 0.024% and a decrease in the distribution width (span) from 3.64 ± 0.013 to 1.26 ± 0.04, which are evident for granules formation by the MADG process. Analysis of variance (ANOVA) (Table 3) revealed that X_1_ and X_2_ had a significant effect on granules’ d_50_ (*p* < 0.0001 and *p* = 0.0002, respectively), with a pronounced impact of X_1_, as indicated by its high sum of squares (45,562.02 for X_1_ and 7816.37 for X_2_). In addition, the d_50_ was positively correlated with X_1_ and X_2_, as evident by the sign of their coefficient estimates (+87.14 for X_1_ and +36.09 for X_2_). Figure 2 shows that the d_50_ significantly increases with increasing both X_1_ and X_2_. Otherwise, X_1_ and X_2_ had a significant (*p* < 0.0001 for X_1_ and *p* = 0.0009 for X_2_) impact on the percent fines, with the highest impact of X_1_, as demonstrated by its higher sum of squares (611.45 for X_1_ and 79.28 for X_2_). Furthermore, values of coefficient of estimation indicated that X_1_ had a marked effect on percent fines in a negative direction, while X_2_ with a low coefficient of estimation value had a low impact in the same direction (−10.10 for X_1_ and −3.36 for X_2_), as shown in Figure 2. This suggests that granulation with a high amount of granulation fluid and long massing time results in producing a low level of fines. Increment of the granulation fluid results in adequate wetting of the particles’ surfaces and buildup of liquid bridges among the particles that improve the granules’ coalescence and growth [13]. In addition, increasing the wet massing time leads to increasing the frequency of particles collision, which improves the granule growth and reduces the percent fines [19]. It was reported that granule growth had been influenced by the mechanical shear of the MADG process [20].

The distribution width (span) determines the broadness of granule size distribution and has a significant effect on granules’ flow, compressibility and segregation [21]. A high value of distribution width indicates a wide size distribution of the system, and vice versa. As shown in Table 3, X_1_ and X_2_ were found to be statistically significant (*p* < 0.0001 and *p* = 0.0008, respectively) with respect to their effect on distribution width, with a prominent impact of X_1_, as evident by its higher sum of squares value (4.05 for X_1_ and 0.6534 for X_2_). The distribution width was found to be negatively correlated with X_1_ and X_2_ according to the sign of their coefficient estimates (−0.8217 for X_1_ and −0.330 for X_2_). As shown in Figure 2, the distribution width was decreased as the granulation fluid and the wet massing time increased.

The generated regression equations that demonstrate the influence of X_1_ and X_2_ on mean granule size, percent fines and distribution width, in terms of coded variables, were expressed as follows:

Mean granule size (µm) = 254.39 + 87.14 × X_1_ + 36.09 × X_2_ (1)

Percent fines (%) = 16.13 − 10.10 × X_1_ − 3.36 × X_2_ (2)

Distribution width = 2.44 − 0.8217 × X_1_ − 0.330 × X_2_ (3)

#### 2.2.2. Granules’ Bulk Density

As indicated in Table 2, increasing the granulation fluid and the wet massing time resulted in increasing the granules’ density from 0.353 ± 0.013 to 0.401 ± 0.006 gcm^−3^. Regression analysis (Table 3) showed that X_1_ and X_2_ had a significant (*p* = 0.0024 and *p* = 0.0007, respectively) impact on granules’ bulk density. However, the influence of X_2_ on bulk density was more pronounced than that of X_1_ according to sum of squares values (0.0005 for X_1_ and 0.0009 for X_2_). Granulation with long wet massing time resulted in exposing the prepared granules to a high shear force for a long period, that led to a decrease of the porosity of granules as well as an increase of the granules’ consolidation and density [22]. The effects of studied variables on granules’ density are shown in Figure 2. The X_2_ had a higher effect on bulk density in a positive direction, while the X_1_ had a low impact in the same direction, as evident by the sign of parameter estimates (+0.0092 for X_1_ and +0.0120 for X_2_). On the other hand, the two-way interaction between X_1_ and X_2_ also had a significant (*p* = 0.0252) impact on bulk density of the prepared granules.

The generated regression equation that demonstrates the influence of X_1_ and X_2_ on bulk density of granules in terms of coded variables was expressed as follows:

Bulk density (gcm^−3^) = 0.3699 + 0.0092 × X_1_ + 0.0120 × X_2_ + 0.0062 × X_1_X_2_ (4)

#### 2.2.3. Granules’ Flow

As displayed in Table 2, the angle of repose decreased from 32.11 ± 0.322° to 26.23 ± 0.415 as the amount of granulation fluid and the wet massing time increased, demonstrating an improvement in powder flow upon granulation using the MADG method. Moravkar et al. reported that the MADG process could produce excellent flowability granules of high drug-loading formulation because of the uniform size of the prepared granules [10]. The results of ANOVA analysis (Table 3) demonstrated that X_1_ and X_2_ had a significant (*p* < 0.0001 and *p* = 0.0002, respectively) negative effect on the angle of repose of prepared granules, as evident by the negative sign of their coefficient estimates (−2.12 for X_1_ and −0.9317 for X_2_). Nevertheless, X_1_ was the predominant variable, as evident by its higher sum of squares (26.92 for X_1_ and 5.21 for X_2_). Further, the two-way interaction between the two variables had a significant (*p* = 0.0356) negative impact on granules’ flow. Figure 2 demonstrates the inverse correlation of tested variables on the angle of repose. As explained above, the MADG process at high amounts of granulation fluid and long massing time resulted in an increased size of granule and bulk density, and a reduced amount of fines. This led to a reduced angle of repose and improved the flow of obtained granules [23].

The generated regression equation that demonstrates the influence of X_1_ and X_2_ on angle of repose of granules in terms of coded variables was expressed as follows:

Angle of repose (degree) = 29.38 − 2.12 × X_1_ − 0.9317 × X_2_ − 0.3325 × X_1_X_2_ (5)

### 2.3. Influence of Process Variables on Tablets’ Properties

#### 2.3.1. Tablet Weight Variation

The main cause for granulation is to provide tablets with acceptable weight variation to assure drug content uniformity, which depends on powder flow [24]. For all runs, average tablet weight and its SD are presented in Table 4. With respect to United States Pharmacopeia (USP) criteria, the variation of tablet weight was acceptable for all runs and the SD was less than 1.9, demonstrating proper granules flow. However, little variation observed in the tablets’ weight was due to variation in granules’ bulk density, as previously discussed in Section 2.2.2. Regression analysis (Table 5) revealed that X_1_ and X_2_ had a significant (*p* < 0.0001 and *p* = 0.004, respectively) negative impact on the SD of tablet weight variation, as indicated from their values of coefficient estimates (−0.1633 for X_1_ and −0.0733 for X_2_). However, X_1_ has a dominant effect due to its higher sum of squares (0.1601 for X_1_ and 0.0323 for X_2_). As shown in Figure 2, both variables have a negative correlation with the SD, indicating that an increase in each variable individually results in a reduction of the SD. In addition, the low SD value was observed in granules prepared at a high level of granulation fluid and long wet massing time, as shown at the higher right corner of the contour plot. This is due to the amelioration of flow of elaborated granules upon increasing the level of granulation fluid and the wet massing time, as previously discussed in Section 2.2.3.

The resulted regression equation that demonstrates the effect of X_1_ and X_2_ on SD of tablet weight variation in terms of coded factors was as follows:

SD of tablet weight variation = 1.66 − 0.1633 × X_1_ − 0.0733 × X_2_ (6)

#### 2.3.2. Tablet Breaking Force and Friability

As the tablet strength is significantly related to the drug release in the patient’s body, it is essential to determine the tablet mechanical strength (i.e., breaking force and friability) [25]. As shown in Table 4, the tablet breaking force decreased from 7.92 ± 0.65 to 6.22 ± 0.23 KP upon the granulation fluid, and the wet massing time increased. ANOVA analysis (Table 5) revealed that X_1_ and X_2_ had a significant (*p* = 0.0004 and *p* = 0.0008, respectively) effect, but nearly equal on tablet breaking force, as evident from values of their sum of squares (1.42 for X_1_ and 1.05 for X_2_). In addition, change in X_1_ and X_2_ had almost the same effect on tablet breaking force in a negative direction, as indicated by the negative sign of their coefficient estimates (−0.4867 for X_1_ and −0.4183 for X_2_). Figure 2 shows that granules prepared at a high level of granulation fluid and long wet massing time produce tablets with a small breaking force. As previously discussed in Section 2.2.2, granulation with a high level of granulation fluid and long wet massing time produced less porous and denser granules with a low fragmentation tendency, which resulted in low tablet breaking force, and vice versa [26]. It was reported that the densified granules prepared by the MADG method might hinder fragmentation, especially when the amount of granulating fluid (water) exceeds 3% [20]. The low compressibility of less porous and high-density granules had been previously reported [22,27].

As shown in Table 4, runs with low levels of granulation fluid (runs 1, 2 and 3) produced friable tablets (i.e., friability > 1.0%). This phenomenon is due to inappropriate wetting of powder particles and formation of high amounts of fines [28]. This finding obviously indicated the inadequacy of these granulation runs for preparing reasonable tablets. On the other hand, granulation runs with high levels of granulation fluid (runs 4–9) produced acceptable tablets with respect to the USP limit (i.e., friability < 1.0%). ANOVA analysis (Table 5) showed that only X_1_ had a significant (*p* < 0.0001 for X_1_ and *p* = 0.2955 for X_2_) effect on friability of the tablet in a negative direction, as evident by the negative sign of coefficient estimates (−0.335 for X_1_ and −0.0367 for X_2_). Figure 2 demonstrates that an increase in the level of granulation fluid leads to a decrease of friability for tablets prepared by the MADG method.

The resulted regression equations that demonstrate the influence of X_1_ and X_2_ on tablet breaking force and friability in terms of coded factors was as follows:

Breaking force (KP) = 7.27 − 0.4867 × X_1_ − 0.4183 × X_2_ (7)

Friability (%) = 0.8589 − 0.335 × X_1_ − 0.0367 × X_2_ (8)

#### 2.3.3. Tablet Disintegration

As shown in Table 4, disintegration time was found to be increased from 9.34 ± 1.75 to 20.29 ± 0.89 min upon the level of granulation fluid, and the wet massing time increased. ANOVA analysis (Table 5) indicated that X_1_ and X_2_ had a significant (*p* < 0.0001 and *p* = 0.0022, respectively) effect on tablet disintegration time in a positive direction, as evident by the positive sign of their coefficient estimates (+4.49 for X_1_ and +1.15 for X_2_). In addition, the two-way interaction of two variables had a significant (*p* = 0.0189) effect in a positive direction (coefficient of estimate = +0.67). Nevertheless, X_1_ had a pronounced impact on disintegration time with respect to the value of its sum of squares (120.87 for X_1_, 7.96 for X_2_ and 1.8 for X_1_ X_2_). Figure 2 indicated that the prolonged disintegration time was associated with a combination of a high level of granulation fluid and long wet massing time, as displayed in the higher right corner of the contour plot. This finding could be attributed to the fact that granulation at high levels of granulation fluid and long wet massing time produce less porous and high-density granules, as previously discussed in Section 2.2.2. These granules retard the penetration of water inside the tablets and delay the disintegration time [28]. This result is in agreement with that previously reported by Takasaki et al. [20]. They reported that the granules prepared by MADG were denser and harder, which hinder fragmentation of the granules and penetration of water into the tablets. Further, a combination of a high amount of granulating fluid and long wet massing time reduced the amounts of fines, which are essential for tablets’ disintegration [29]. Takasaki et al. reported that increasing the water amount delayed the disintegration time of tablets prepared by the MADG process [12].

The resulted regression equation that demonstrates the effect of X_1_ and X_2_ on tablet disintegration time in terms of coded factors was expressed as follows:

Disintegration time (min) = 11.96 + 4.49 × X_1_ + 1.15 × X_2_ + 0.67 × X_1_ X_2_ + 2.0× X_1_^2^ + 0.1417 × X_2_^2^ (9)

#### 2.3.4. Tablet Dissolution

**Figure 3:**
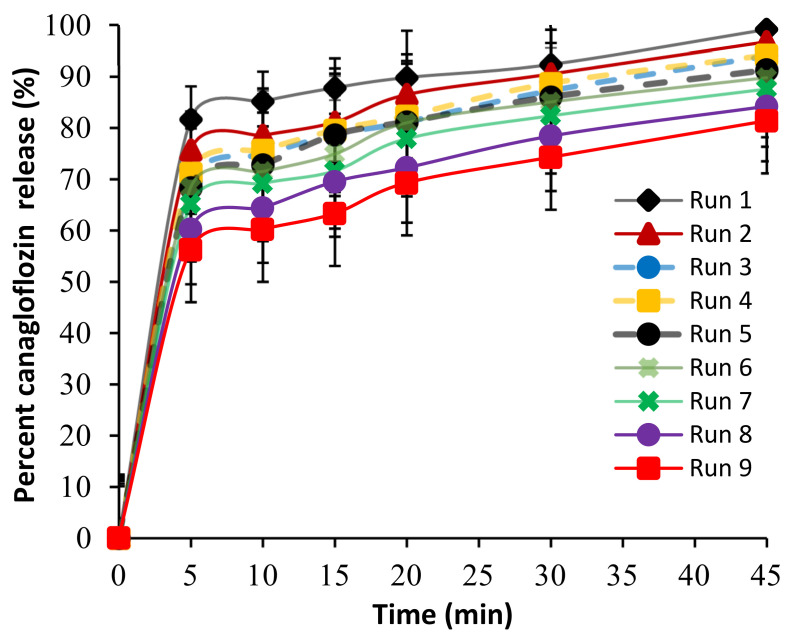
Release profiles of canagliflozin tablets according to 3^2^ full-factorial design.

Tablet dissolution is a key factor as it controls the drug release from the tablet, and therefore its bioavailability [30]. Release profiles of CNG tablets are presented in Figure 3. According to the USP standards for immediate release tablets, all prepared tablets showed acceptable drug release (i.e., 85% release within 30 min) except runs 7, 8 and 9 which released 82.32% ± 3.97%, 78.35% ± 4.61% and 74.21% ± 4.15% respectively, after 30 min (Table 4 and Figure 3). Regression analysis (Table 5) revealed that X_1_ and X_2_ had a significant (*p* = 0.0002 and *p* = 0.007, respectively) negative effect on drug release, as evident by the negative sign of their coefficient of estimates (−5.86 for X_1_ and −2.81 for X_2_). However, X_1_ was the most important variable, as shown by its higher sum of squares compared to X_2_ (206.27 for X_1_ and 47.38 for X_2_). Figure 2 showed that both variables were inversely proportional with the percentage of drug release after 30 min. In addition, formulations prepared at a combination of a low level of granulation fluid and short wet massing time showed faster release than others. This could be attributed to the fact that granulation at low levels of granulation fluid and short wet massing time produce small size and low-density granules, as previously described, which rapidly eroded, disintegrated and rapidly released the drug [27].

The resulted regression equation that demonstrates the effect of X_1_ and X_2_ on percentage of drug release after 30 min in terms of coded factors was expressed as follows:

Drug release after 30 min (%) = 84.96 − 5.86 × X_1_ − 2.81 × X_2_ (10)

### 2.4. Optimization of Process Variables Using Desirability Function

**Figure 4:**
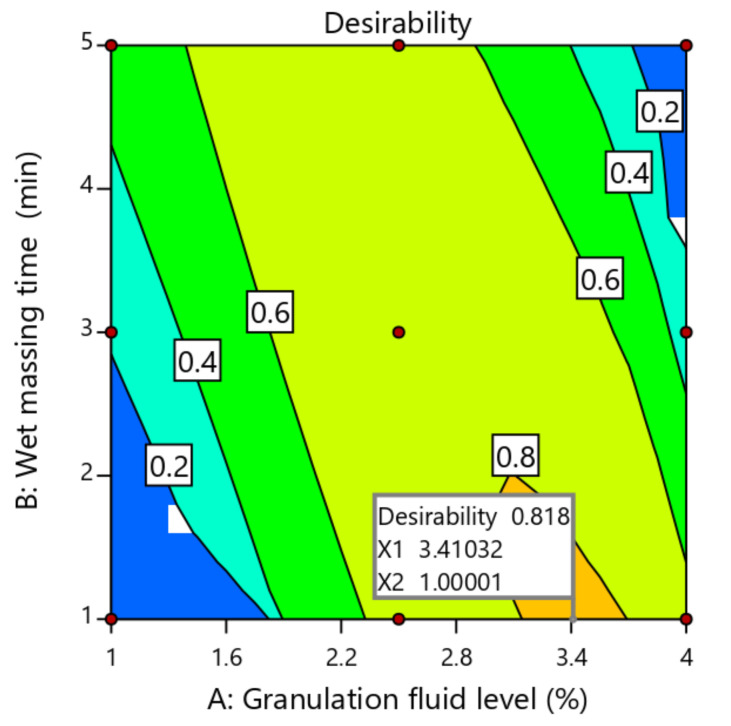
Contour plot showing the levels of optimized process variables and overall desirability.

The main purpose of the optimization step was to optimize the process variables to produce products with desired properties [25]. As shown in Table 6, numerical optimization using the desirability function was done by setting goals for each dependent response. For successful CNG tablet formulation, acceptable weight variation, mechanical strength, disintegration time and percent release with respect to USP standards are required. It was expected that the independent variables that produce CNG tablets that complied with the USP standards would be 3.41% w/w and 1.0 min for granulation fluid and wet massing time respectively, with an overall desirability of 0.818 (Figure 4). As presented in Table 7, the observed values of breaking force (7.15 ± 1.83 KP), friability (0.71 ± 0.95 %), disintegration time (13.56 ± 0.76 min) and drug release at 30 min (87.25 ± 2.13 %) were in close agreement with the predicted values of breaking force (7.39 KP), friability (0.69%), disintegration time (14.0 min) and drug release at 30 min (84.21%). In addition, the small value of calculated relative errors (<5.0%) assured the validity of the applied design.

## 3. Materials and Methods

### 3.1. Materials

Canagliflozin was kindly donated by JPI Ltd. Co. (Al-kharj, Saudi Arabia). Polyvinylpyrrolidone (Kollidon^®^ 25 (BASF Co., Ludwigshafen, Germany)), microcrystalline cellulose (Avicel^®^ PH-200 (DuPot Inc. Co., Philadelphia, PA, USA)), colloidal silicon dioxide (Aerosil 200^®^ (Evonic, Hanau-Wolfgang, Germany)), croscarmellose sodium (Vivasol^®^ (JRS pharma, Rosenberg, Germany)) and magnesium stearate (Sinwon chemical Co., Siheung, Korea) were used as received.

### 3.2. Experimental Design

Before generation of the design, preliminary studies were performed to determine the independent variables and ranges of each variable at which proper granules and tablets were obtained. The 3^2^ full-factorial design was done to explore the influence of two process variables: granulation fluid level (X_1_) and wet massing time (X_2_), on the critical quality attributes (CQAs) of granules and tablets using Design-Expert^®^ software Version-11 (State-ease, Inc. Minneapolis, USA). Each variable was evaluated at three levels, coded as −1, 0 and +1, for low, medium and high, respectively (Table 8). The full matrix of the generated design is shown in Table 9. The run at the center point was performed in triplicate to validate the design and prevent the experimental error. The ANOVA test using Design-Expert software was applied to investigate the influence of independent variables on the studied dependent responses at the 95% level of significance. In order to suggest the significance of the selected model, the *R*^2^ and *p*-value (should be <0.05) of the proposed models were compared. The general polynomial equation applied for the 3^2^ factorial design is as follows:

Response variable (Y) = β_0_ + β_1_ X_1_ +β_2_ X_2_ + β_3_ X_1_X_2_ + β_4_ X_1_^2^ + β_5_ X_2_^2^ (11)

where β_0_ is the arithmetic mean response of all runs, and β_1_, β_2_, β_3_, β_4_ and β_5_ are regression coefficients of estimate of the independent variables X_1_ and X_2_. X_1_ X_2_ and X_1_^2^ and X_2_^2^ represent the interaction and quadratic effect, respectively. The relative error was determined using the following formula [31]:

(12) $$\mathrm{Relative}\;\mathrm{error}\;\left(\%\right)=\left(\;\frac{\left|\mathrm{Predicted}\;\mathrm{value}-\mathrm{Experiment}\;\mathrm{value}\right|}{\mathrm{Predicted}\;\mathrm{value}}\;\right)\times \;100$$

### 3.3. Manufacture of Granules and Tablets

The formulation used in the current study was shown in Table 10. MADG runs (the batch size was 400 g) were performed in a high-shear mixer/granulator (BOSCH Packaging Technology, Schopfheim, Germany). The specified amount of CNG and polyvinylpyrrolidone were dry-blended in the high-shear mixer for two minutes at high speeds of impeller and chopper (300 and 2000 rpm, respectively). The produced blend was then granulated by addition of a very small amount of granulation fluid (1–4% *w*/*w*) using a binary spray nozzle. Distilled water was used as granulation fluid in the current study. Following water addition, the blend was wet massed for a particular wet massing time according to the experimental design. For the two min absorption stage, the chopper was stopped and absorbent materials (i.e., microcrystalline cellulose and colloidal silicon dioxide) were added. Finally, disintegrant croscarmellose sodium and pre-sieved lubricant magnesium stearate were directly added to the blend and mixed in the granulator for 2.0 min and 1.0 min respectively, at low impeller speed (200 rpm). The final blend was then compressed into 400 mg tablets at a compression force of 13 KN using a rotary tablet press (RoTap-T 2.0, Kg pharma, Berlin, Germany). The machine was adjusted to provide four tablets per run using 10 mm flat tablet tooling. The obtained tablets were collected for further investigation.

### 3.4. Evaluation of Prepared Granules

#### 3.4.1. Mean Granule Size (d_50_)

Mean granule size of the prepared granules was measured using the dry dispersion technique of the laser diffraction particle size analyzer (Mastersizer 2000, Malvern Instruments Ltd., Worcestershire, UK). Approximately 5–6 g samples were air-dispersed at an inlet air pressure of one bar and a feed-rate of 30%. Obscuration was adjusted between 0.6% and 6%.

#### 3.4.2. Granules’ Bulk Density

Bulk density (ρ_b_) was measured by carefully pouring the granules into a 50 cm^3^ graduated cylinder. Bulk volume (Vb) of the granules sample and corresponding mass (M) were determined. The ρ_b_ was determined using Equation (13) [32]:

(13) $$\rho_{b}\;=\;\frac{M}{\mathrm{Vb}}$$

#### 3.4.3. Granules Flow

The granules flow was determined using the static angle of repose procedure. A dry funnel was clamped at 2 cm (H) above a clean paper placed on a flat surface. The granules were carefully poured through the dry funnel until the apex of the cone, just reaching the tip of the funnel. The average diameters (D) of the cone base were determined and the angle of repose was determined using Equation (14) [32]:

(14) $$\tan\;\left(\theta \right)\;=\;\frac{2H}{D}$$

### 3.5. Evaluation of Prepared Tablets

#### 3.5.1. Weight Variation

Individual weight of twenty randomly selected tablets was determined using an analytical balance (Mettler Toledo New Classic ML204/01, Ohio, OH, USA). The weight variation was assessed by considering the standard deviation (SD) of tablet weight. Results are presented as mean ± SD.

#### 3.5.2. Breaking Force

Individual breaking force (hardness) for ten randomly selected tablets was measured using an automatic hardness tester (Erweka Multi-Check 5.1, Heusenstamm, Germany). Results are presented as mean ± SD.

#### 3.5.3. Friability

Friability of prepared tablets was done according to the method mentioned in USP [32]. Ten randomly selected tablets were accurately weighed (W_1_) using an analytical balance (Mettler Toledo New Classic ML204/01, Ohio, OH, USA) and placed in a friability tester (Toyama Sangyo TFT-1200, Osaka, Japan), rotated at 25 rpm for 4 min. The tablets were de-dusted and accurately weighed (W_2_). Friability was calculated using Equation (15):

(15) $$\mathrm{Friability}=\;\frac{W1-W2}{W1}\;\times \;100$$

#### 3.5.4. Tablet Disintegration

Disintegration of prepared tablets was carried out according to the method described in USP [32]. The in vitro disintegration test for six randomly selected tablets was done using a disintegration tester (Erweka, ZT4, Heusentsamn, Germany) in 800 mL of distilled water kept at 37 ± 0.5 °C. For each tablet, disintegration time was recorded in minutes when all solids passed through the screen of the disintegration apparatus. Results are presented as mean ± SD.

#### 3.5.5. Tablet Dissolution

The drug release for six randomly selected tablets was performed using a dissolution tester (Distek 2500, Distek Inc., New Jersey, NJ, USA) following the USP paddle method [32]. The test was done in 900 mL of pH 6.8 phosphate buffer solution (with 0.75% *w*/*v* sodium lauryl sulphate) kept at 37 ± 0.5 °C with a paddle rotation speed at 75 rpm. After specified time intervals of 5, 10, 15, 20, 30 and 45 min, samples of dissolution medium were analyzed using in situ fiber optic UV testing (Distek Opt-Dis 410, Distek Inc., New Jersey, NJ, USA) at λ_max_ of 290 nm [5,33].

## 4. Conclusions

MADG showed good opportunities to address the problems of poor flow and tableting problems of canagliflozin, which is critical for effective development of small size tablets of high-loading drugs, and consequently, improved the patient compliance due to ease of tablet swallowing. Quantitative correlation between MADG key process variables, intermediate granules and final product tablets have been established using the design of experiment approach. In particular, regression analysis of obtained data demonstrated the significant (*p* ≤ 0.05) effect of tested process variables on properties of granules and corresponding tablets, with a pronounced impact of the granulation fluid level. The levels of optimized process conditions that produce good flow granules and acceptable tablets were high level of granulation fluid (3.41% *w*/*w*) and short wet massing time (1.0 min). From an industrial perspective, application of the MADG technique can significantly decrease the manufacturing cost, as obtained granules could be directly compressed into tablets without drying and milling. In summary, this study increased knowledge of the influence of key process variables of MADG on properties of granules and tablets and could serve as a backbone for further developing a mechanistic model for the MADG process.

## Figures and Tables

**Table 1:** Model summary statistics of dependent responses.

Response	Model	*p*-Value	*R* ^2^	Adjusted *R*^2^	Predicted *R*^2^
Mean granule size (d_50_)	Linear	<0.0001	0.9872	0.9830	0.9700
Percent fines	Linear	<0.0001	0.9816	0.9755	0.9621
Distribution width	Linear	<0.0001	0.9787	0.9716	0.9480
Bulk density	* 2FI	0.0011	0.9510	0.9217	0.8011
Angle of repose	2FI	<0.0001	0.9917	0.9868	0.9558
** SD of weight variation	Linear	0.0001	0.9531	0.9375	0.8913
Breaking force	Linear	0.0002	0.9381	0.9175	0.8353
Friability	Linear	0.0001	0.9487	0.9316	0.8565
Disintegration time	Quadratic	0.0003	0.9982	0.9953	0.9809
Drug release at 30 min	Linear	0.0003	0.9350	0.9134	0.8447

* 2FI: two factor interaction model and ** SD: standard deviation.

**Table 2:** Physical properties of prepared granules (mean ± SD).

Runs	Mean Granule Size (d_50_) (µm ± SD)	Percent Fines (% ± SD)	Distribution Width (span)	Bulk Density (gcm^−3^ ± SD)	Angle of Repose (Degree ± SD)
1	117.12 ± 0.25	28.08 ± 0.013	3.64 ± 0.013	0.353 ± 0.013	32.11 ± 0.322
2	184.33 ± 0.21	26.51 ± 0.034	3.42 ± 0.023	0.365 ± 0.002	31.35 ± 0.535
3	206.11 ± 0.31	22.46 ± 0.012	2.74 ± 0.021	0.367 ± 0.025	31.13 ± 0.026
4	222.17 ± 0.26	20.61 ± 0.027	2.65 ± 0.022	0.357 ± 0.023	30.51 ± 0.411
5	246.32 ± 0.29	18.33 ± 0.037	2.41 ± 0.041	0.371 ± 0.006	29.23 ± 0.243
6	283.05 ± 0.34	12.72 ± 0.015	2.25 ± 0.036	0.376 ± 0.038	28.21 ± 0.334
7	312.45 ± 0.66	10.31 ± 0.024	1.94 ± 0.015	0.362 ± 0.009	28.54 ± 0.212
8	338.82 ± 0.34	4.16 ± 0.028	1.67 ± 0.034	0.377 ± 0.072	27.11 ± 0.722
9	379.14 ± 0.33	2.01 ± 0.024	1.26 ± 0.048	0.401 ± 0.006	26.23 ± 0.415

**Table 3:** Regression analysis of dependent responses of prepared granules.

Variables	Coefficient Estimate	Sum of Squares	Standard Error	*F*-Value	*p*-Value	95 % CI * Low	95 % CI High
**Mean granule size (d_50_)** **(linear model)**							
Intercept	254.39	-	3.58	-	-	245.64	263.14
X_1_	87.14	455,562.02	4.38	395.83	**<0.0001**	76.42	97.86
X_2_	36.09	7816.36	4.38	67.91	**0.0002**	25.38	46.81
**Percent fines** **(linear model)**							
Intercept	16.13	-	0.4897	-	-	14.93	17.33
X_1_	−10.10	611.45	0.5997	283.33	**<0.0001**	−11.56	−8.63
X_2_	−3.63	79.28	0.5997	36.74	**0.0009**	−5.10	−2.17
**Distribution width** **(linear model)**							
Intercept	2.44	-	0.0436	-	-	2.34	2.55
X_1_	−0.8217	4.05	0.0534	237.03	**<0.0001**	−0.9523	−0.6911
X_2_	−0.3300	0.6534	0.0534	38.23	**0.0008**	−0.4606	−0.1994
**Bulk density** **(2FI model)**							
Intercept	0.3699	-	0.0013	-	-	0.3665	0.3733
X_1_	0.0092	0.0005	0.0016	32.12	**0.0024**	0.0050	0.0133
X_2_	0.0120	0.0009	0.0016	55.05	**0.0007**	0.0078	0.0162
X_1_ X_2_	0.0062	0.0002	0.0020	9.96	**0.0252**	0.0012	0.0113
**Angle of repose** **(2FI model)**							
Intercept	29.38	-	0.0777	-	-	29.18	29.58
X_1_	−2.12	26.92	0.0951	496.13	**<0.0001**	−2.36	−1.87
X_2_	−0.9317	5.21	0.0951	95.97	**0.0002**	−1.18	−0.6872
X_1_ X_2_	−0.3325	0.4422	0.1165	8.15	**0.0356**	−0.6319	−0.0331

X_1_ and X_2_ are the level of granulation level and the wet massing time, respectively. X_1_X_2_ is the effect of interaction. * CI: confidence interval.

**Table 4:** Physical properties of prepared canagliflozin tablets (mean ± SD).

Runs	Weight (mg ± SD)	Thickness (mm ± SD)	Breaking Force (KP ± SD)	Friability (% ± SD)	Disintegration Time (min ± SD)	% Release after 30 min (% ± SD)
1	399.55 ± 1.86	3.31 ± 0.014	7.92 ± 0.65	1.30 ± 0.03	9.34 ± 1.75	92.32 ± 3.15
2	397.83 ± 1.80	3.33 ± 0.006	7.89 ± 0.15	1.26 ± 0.11	9.27 ± 1.21	90.53 ± 2.15
3	400.91 ± 1.79	3.36 ± 0.005	7.31 ± 0.66	1.03 ± 0.06	10.09 ± 1.22	87.21 ± 3.34
4	399.71 ± 1.78	3.35 ± 0.03	7.89 ± 0.78	0.82 ± 0.02	10.65 ± 2.67	88.66 ± 2.55
5	399.82 ± 1.61	3.34 ± 0.004	7.33 ± 0.98	0.88 ± 0.04	12.14 ± 1.43	86.01 ± 2.97
6	398.15 ± 1.60	3.36 ± 0.007	6.89 ± 0.93	0.86 ± 0.05	13.38 ± 1.54	85.02 ± 3.88
7	398.35 ± 1.59	3.33 ± 0.006	7.12 ± 0.39	0.53 ± 0.01	16.86 ± 2.59	82.32 ± 3.97
8	397.61 ± 1.48	3.32 ± 0.005	6.86 ± 0.59	0.51 ± 0.02	18.47 ± 1.54	78.35 ± 4.61
9	401.22 ± 1.40	3.33 ± 0.03	6.22 ± 0.23	0.54 ± 0.01	20.29 ± 0.89	74.21 ± 4.15

**Table 5:** Regression analysis of dependent responses of CNG tablets.

Variables	Coefficient Estimate	Sum of Squares	Standard Error	*F*-Value	*p*-Value	95 % CI Low	95 % CI High
**SD of weight variation** **(linear model)**							
Intercept	1.66	-	0.0132	-	-	1.62	1.69
X_1_	−0.1633	0.1601	0.0162	101.45	**<0.0001**	−0.2030	−0.1237
X_2_	−0.0733	0.0323	0.0162	20.45	**0.0040**	−0.1130	−0.0337
**Breaking force** **(linear model)**							
Intercept	7.27	-	0.0549	-	-	7.14	7.40
X_1_	−0.4867	1.42	0.0673	52.34	**0.0004**	−0.6513	−0.3221
X_2_	−0.4183	1.05	0.0673	38.67	**0.0008**	−0.5829	−0.2537
**Friability** **(linear model)**							
Intercept	0.8589	-	0.0261	-	-	0.7949	0.9228
X_1_	−0.3350	0.06734	0.0320	109.57	**<0.0001**	−0.4133	−0.2567
X_2_	−0.0367	0.0081	0.0320	1.31	0.2995	−0.1150	0.0416
**Disintegration time** **(Quadratic model)**							
Intercept	11.96	-	0.2135	-	-	11.28	12.64
X_1_	4.49	120.87	0.1169	1471.88	**<0.0001**	4.11	4.86
X_2_	1.15	7.96	0.1169	96.98	**0.0022**	0.7795	1.52
X_1_X_2_	0.6700	1.80	0.1432	21.88	**0.0185**	0.2142	1.12
X_1_^2^	2.00	7.97	0.2026	97.17	**0.0022**	1.35	2.64
X_2_^2^	0.1417	0.0401	0.2026	0.4891	**0.5347**	−0.5030	0.7863
**Percent release at 30 min** **(linear model)**							
Intercept	84.96	-	0.5714	-	-	83.56	86.36
X_1_	−5.86	206.27	0.6998	70.21	**0.0002**	−7.58	−4.15
X_2_	−2.81	47.38	0.6998	16.12	**0.0070**	−4.52	−1.10

X_1_ and X_2_ are the granulation fluid level and the wet massing time, X_1_X_2_ is the effect of interaction and X_1_^2^ and X_2_^2^ are the sum of effects.

**Table 6:** The constraints adopted for optimization of process variables and determination of overall desirability.

Variables	Target	Range	Weight	Importance Coefficient
**Input**				
* Granulation fluid	In range	1–4%	1	−
Wet massing time	In range	1–5 min	1	−
**Output**				
SD of weight variation	In range	1.4–1.86	1	−
Breaking force	In range	6.22–7.92%	1	−
Friability	0.6%	0.51–1.3%	1	++++ ^#^
Disintegration time	10 min	9.27–20.29 min	1	+++++ ^#^
Percent release at 30 min	85%	74.21–92.32%	1	+++++ ^#^

* Distilled water was used as granulation fluid, − no value and ^#^ means level of importance.

**Table 7:** Predicted and observed (actual) values for dependent responses of optimized run with their relative errors.

Responses	Predicted Values	Observed Values (Mean ± SD)	Relative Error (%)
SD of weight variation	1.63	1.58	3.06
Breaking force (KP)	7.39	7.15 ± 1.83	3.24
Friability (%)	0.69	0.71 ± 0.95	−2.89
Disintegration time (min)	14.00	13.56 ± 0.76	3.14
Percent release at 30 min	84.21	87.25 ± 2.13	−3.61

**Table 8:** The levels of independent process variables used in design of experiment.

Coded Levels	Granulation Fluid * (%)	Wet Massing Time (min)
−1	1	1
0	2.5	2
1	4	3

−1: variable at low level, 0: variable at medium level, 1: variable at high level. * Water was used as granulation fluid.

**Table 9:** A 3^2^ full-factorial design of independent process variables.

Run	Granulation Fluid * (% *w*/*w*)	Wet Massing Time (min)
1	1	1
2	1	2
3	1	3
4	2.5	1
5	2.5	2
6	2.5	3
7	4	1
8	4	2
9	4	3

* Water was used as granulation fluid.

**Table 10:** The quantitative composition of canagliflozin tablet formulation.

Ingredients	% *w*/*w*
Canagliflozin	75
Polyvinylpyrrolidone (K25)	10
Sodium starch glycolate	4
Colloidal silicon dioxide	1.5
Microcrystalline cellulose	8.5
Magnesium stearate	1
